# A Comparative Analysis of Prophylactic Antibiotic Administration in Emergency Surgery Versus Elective Surgery: A Comprehensive Review

**DOI:** 10.7759/cureus.57338

**Published:** 2024-03-31

**Authors:** Christopher R Meretsky, Brandon Krumbach, Anthony T Schiuma

**Affiliations:** 1 Surgery, St. George's University School of Medicine, Great River, USA; 2 Surgery, St. George’s University School of Medicine, Great River, USA; 3 Orthopedic Surgery, Holy Cross Hospital, Fort Lauderdale, USA

**Keywords:** antibiotic administration, emergency and elective surgery, general emergency surgery, postoperative complication, preoperative planning, prophylactic antibiotics, surgical site complication, surgical site infection (ssi)

## Abstract

Surgical site infections (SSIs) contribute to increased patient morbidity, prolonged hospital stays, and substantial healthcare costs. Prophylactic antibiotics play a pivotal role in mitigating the risk of SSIs, with their administration being a standard practice before both emergency and elective surgeries. This paper provides a comprehensive review and comparative analysis of the benefits of prophylactic antibiotic administration in emergency surgery versus elective surgery. Through a systematic literature review and analysis of relevant studies identified through PubMed searches, this paper highlights the specific benefits of prophylactic antibiotics between emergency and elective surgeries. The findings underscore the importance of tailored antibiotic regimens and administration protocols to optimize patient care and promote successful surgical outcomes in diverse clinical settings. Further research is warranted to refine guidelines and enhance understanding of the relationship between prophylactic antibiotics and surgical outcomes across different surgical contexts.

## Introduction and background

Introduction

Surgical site infections (SSIs) present a significant burden on patients and healthcare systems worldwide, both in terms of patient outcomes and economic costs [1-3].

To be classified as an SSI, the infection must appear within 30 days post-surgery (or within one year for organ or space infections with an implant), involve either the skin, subcutaneous tissues, deep layers, or distant organs, and exhibit either purulent drainage or the presence of organisms isolated from the wound site [4]. The initial stage in the formation of an SSI involves microbial contamination of the wound, stemming from either endogenous or exogenous origins. Symptoms commonly observed in SSIs comprise redness, localized pain, prolonged fever without a clear cause, discharge from the wound often containing pus, wound opening, and difficulties with wound healing [4].

SSIs can cause significant patient morbidity, extend hospital stays, and, in severe instances, lead to mortality. Additionally, SSIs might necessitate further surgical interventions, such as debridement or implant extraction, for effective infection management. Consequently, these infections pose a significant financial strain on healthcare systems due to heightened hospitalization expenses, prolonged antibiotic treatments, and potential legal ramifications [5,6]. As a result, reducing SSIs enhances patient outcomes and promotes cost-efficient healthcare. Therefore, the implementation of robust preventive strategies is imperative for minimizing the occurrence of SSIs.

Among these strategies, the administration of prophylactic antibiotics stands as a cornerstone in preoperative care protocols. By targeting potential pathogens before incision, prophylactic antibiotics aim to reduce the bacterial load at the surgical site and subsequently lower the risk of SSIs [3]. Effective administration of antibiotics at predetermined time increments has led to a notable decrease in the occurrence of SSIs. Nevertheless, utilizing antibiotics for prophylaxis presents challenges, such as an increased risk of antibiotic resistance and potential adverse effects. There is a need for continuous evaluation and refinement of antibiotic prophylaxis protocols to enhance patient safety and improve surgical outcomes.

This paper explores the distinctions in the benefits of prophylactic antibiotics between emergency and elective surgeries, providing a nuanced understanding of their respective roles in infection prevention.

Emergency surgery and prophylactic antibiotics

Emergency surgeries, characterized by time constraints and unpredictable clinical scenarios, benefit significantly from the use of prophylactic antibiotics. These benefits include infection prevention in high-risk scenarios and tailored antibiotic selection. Firstly, the rapid initiation of prophylactic antibiotics is feasible in emergency settings, allowing for prompt administration even when preoperative planning is limited. This intervention holds the potential to minimize the risk of SSIs amidst the urgency of the procedure. Secondly, in high-risk scenarios such as trauma or acute conditions necessitating emergent surgeries, the heightened risk of infection underscores the importance of prophylactic antibiotics. By reducing this risk, prophylactic antibiotics contribute to improved patient outcomes in these critical situations. Lastly, antibiotic selection is crucial in emergency surgeries, where broad-spectrum antibiotics covering a wide range of potential pathogens may be preferred. This approach addresses the uncertainty surrounding the surgical site and the patient's medical history, enhancing the effectiveness of prophylactic antibiotic therapy in preventing SSIs during emergency procedures.

Elective surgery and prophylactic antibiotics

On the other hand, elective surgeries present distinct advantages for the administration of prophylactic antibiotics. These benefits include enhanced precision in timing, tailored antibiotic regimens, and a more controlled environment for administration. In particular, optimal timing can be achieved as these procedures allow for thorough preoperative planning, facilitating the timely administration of prophylactic antibiotics for optimal serum and tissue concentrations. Moreover, elective surgeries offer the opportunity for a tailored approach to antibiotic regimens, taking into account the specific procedure, patient characteristics, and potential pathogens, unlike the urgency often associated with emergency surgeries. Also, the reduced urgency for administration in elective surgeries allows healthcare providers to administer prophylactic antibiotics more precisely, ensuring optimal patient care and minimizing the risk of SSIs and other complications.

In addition to these benefits, the use of prophylactic antibiotics in both emergency and elective surgeries can also lead to reduced postoperative complications, shorter hospital stays, and lower healthcare costs [7]. Studies have shown that administering prophylactic antibiotics before surgery significantly decreases the incidence of SSIs and other surgical complications, ultimately improving patient outcomes and reducing the burden on healthcare systems. Moreover, the judicious use of antibiotics in emergency settings can help combat the rise of antibiotic resistance, ensuring that these life-saving medications remain effective for future patients. Therefore, the incorporation of prophylactic antibiotics into emergency surgical protocols is essential for optimizing patient care and promoting successful surgical outcomes.

## Review

Comparative analysis

We compared the benefits of prophylactic antibiotics in emergency and elective surgeries based on available literature, considering infection rates, postoperative complications, length of hospital stay, and healthcare costs. This comparative analysis aims to provide valuable insights into the effectiveness of prophylactic antibiotic use in different surgical contexts, ultimately informing clinical decision-making and healthcare policy development.

Research strategy

**Figure 1:**
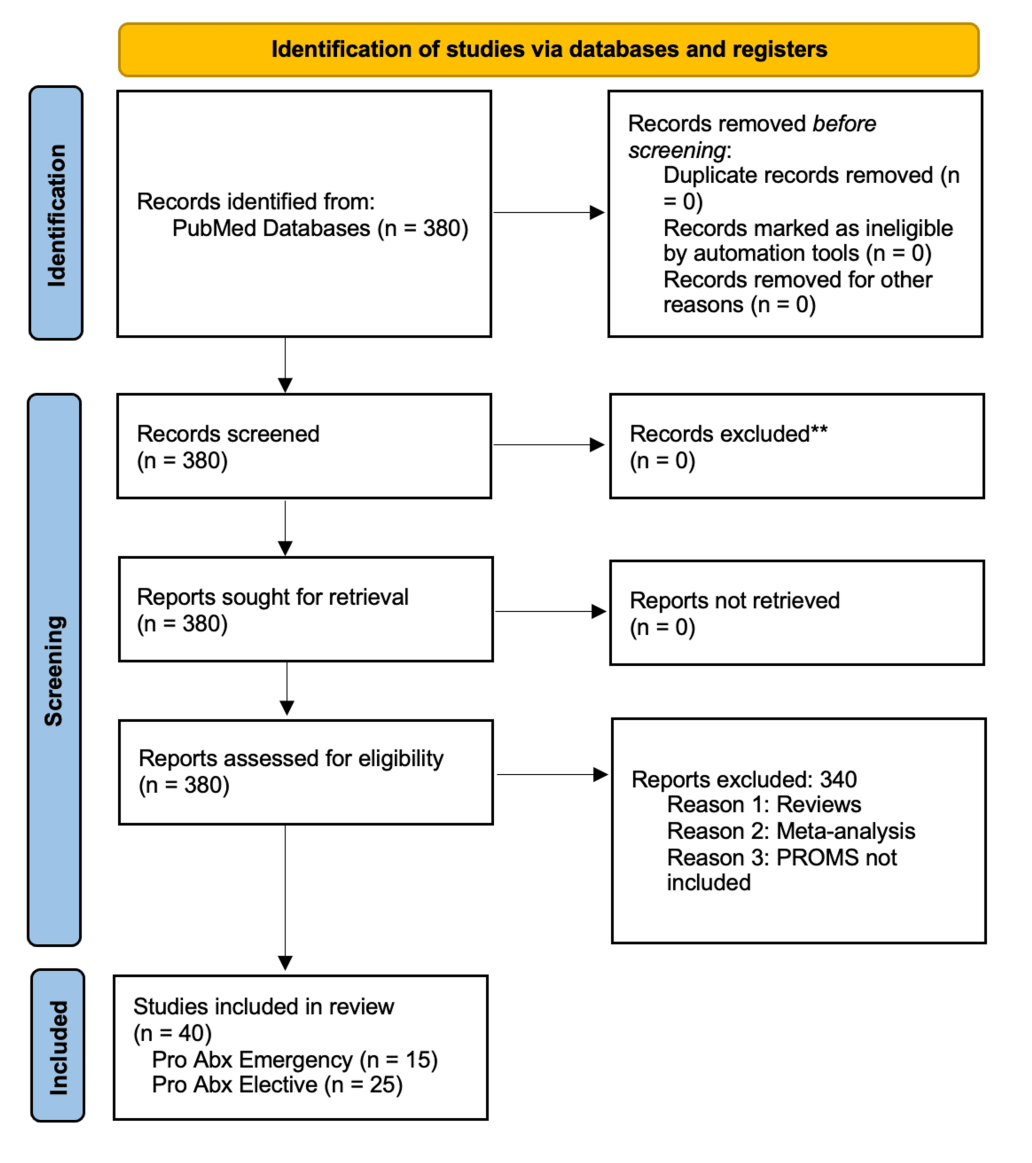
PRISMA flowchart: literature search and study selection Following the PRISMA guidelines, our search was conducted using the academic database PubMed, with relevant keywords. Our search was focused on the outcomes of prophylactic antibiotics across multiple surgical sub-specialties, with a focus on elective and emergency. We included relevant studies published over a 20-year period (2004-2024) that met the inclusion criteria of this review paper. n: number, PRISMA: Preferred Reporting Items for Systematic Reviews and Meta-Analyses, Pro: prophylactic, Abx: antibiotics

This analysis involved a comprehensive review of existing literature related to the benefits of prophylactic antibiotics in emergency and elective surgeries. Two literature searches were conducted using the academic database PubMed, with relevant keywords. The first study search included keywords such as "prophylactic antibiotics," "surgical site infections," and "emergency surgery," while the second one included "prophylactic antibiotics," "surgical site infections," and "elective surgery" over a 20-year period (2004-2024), in line with the Preferred Reporting Items for Systematic Reviews and Meta-Analyses (PRISMA) guidelines. Articles published in peer-reviewed journals and relevant medical guidelines were included in the review. The study selection process is illustrated in Figure 1.

The analysis focused on comparing the benefits of prophylactic antibiotics in emergency and elective surgeries across several key factors, including infection rates, patient outcomes, healthcare resource utilization, length of hospital stay, and healthcare costs. Each factor was evaluated based on the findings reported in the literature, with particular attention to studies that directly compared outcomes between emergency and elective surgeries.

Data synthesis involved summarizing the findings from the selected studies and identifying common themes or patterns across different research articles. Any discrepancies or conflicting findings were noted, and efforts were made to reconcile the differences through further examination of the literature.

Results

Prophylactic Antibiotics in Emergency Surgery

A PubMed search conducted within the last 20 years (2004-2024), using the keywords "prophylactic antibiotics," "surgical site infections," and "emergency surgery," yielded 165 results. Among these, 15 articles were selected because they met the criteria of being clinical trials or randomized controlled trials, while the remaining were excluded because they were reviews [8-22].

The effectiveness of prophylactic antibiotics was assessed across various surgical procedures, revealing mixed findings. In obstetric surgeries, such as cesarean sections, prophylactic antibiotic administration has been shown to significantly reduce the risk of postoperative infections, including SSIs [9,11,14,16].

One randomized controlled trial comparing single-dose versus multiple-dose antibiotic prophylaxis for preventing cesarean section postpartum infections demonstrated that intravenous ceftriaxone (1 g) and metronidazole (500 mg) administered 30-60 minutes before incision, with additional parenteral doses for 48 hours and subsequent oral cefuroxime 500 mg every 12 hours and metronidazole 400 mg every eight hours for five days, resulted in a statistically significant reduction in clinical endometritis (0.0% vs. 6.1%, p = 0.028) when compared to single-dose administration [9]. Moreover, while no significant difference was observed in the incidence of wound infection and febrile morbidity between single-dose and multiple-dose prophylaxis, single-dose prophylaxis resulted in lower rates of clinical endometritis, suggesting its cost-effectiveness [9]. Similarly, a clinical trial conducted at Mulago Hospital in Uganda demonstrated a lower risk of overall postoperative infection (relative risk (RR) 0.77, 95% CI 0.62-0.97) and reduced incidence of endometritis (RR 0.62; 95% CI 0.39-0.99; p = 0.036) when prophylactic antibiotics were administered within an hour before incision [14]. In addition, a randomized equivalence-controlled trial at Bugando Medical Centre in Tanzania compared a single dose of gentamicin in combination with metronidazole to multiple doses for preventing post-cesarean infection, revealing SSI rates of 4.8% and 6.4%, respectively, with an absolute proportion difference of 1.6% (95% CI: -2.4 - 5.6%), suggesting non-equivalence between the two dosing regimens [16].

In studies focusing on non-obstetric emergency surgeries, such as laparoscopic cholecystectomy for acute cholecystitis, the use of prophylactic antibiotics did not significantly impact the incidence of infectious complications [8]. For example, a double-blind, placebo-controlled, randomized trial investigating the clinical significance of preoperative antibiotics in mild to moderate acute cholecystitis patients undergoing emergency laparoscopic cholecystectomy found that the postoperative infection rate was 8.6% in the antibiotic group and 7.6% in the placebo group, with no statistically significant difference observed (absolute difference, 1%; 95% CI: −8.1% to 6.1%; p = 0.815) [8]. Moreover, based on a noninferiority margin of 10%, the absence of preoperative antibiotic treatment was not associated with worse clinical outcomes than antibiotic treatment, suggesting that prophylactic antibiotics may not be necessary in such cases [8].

Another study showed that in cases such as facial lacerations caused by dog bites, immediate primary closure has been shown to promote primary healing without increasing the risk of infection [15]. In this study, 600 cases were randomly divided into two groups: group A had their facial lacerations left open after thorough debridement, while group B underwent immediate primary closure. Antibiotics were only used if the wound became infected, not prophylactically. The infection rate for groups A and B was 8.3% and 6.3%, respectively, with no significant difference between them. The healing time was significantly shorter in group B compared to group A for both untainted and infected cases. These findings suggest that immediate primary closure of facial lacerations from dog bites can shorten the healing time without increasing the risk or duration of infection, making prophylactic antibiotics unnecessary [15].

On the other hand, in patients with acute calculous cholecystitis, using antibiotic prophylaxis has been demonstrated to be more effective in preventing SSIs. For example, a multicenter, randomized, open-label, non-inferiority clinical trial assigned adults with mild-to-moderate acute calculous cholecystitis to receive either 2 g of cefazolin administered before incision or no antibiotic prophylaxis [10]. The primary goal was to see how many patients developed infections within 30 days after surgery. Results revealed that 7.1% of patients who received antibiotic prophylaxis had infections, compared to 12.6% in the group without prophylaxis, with an absolute difference of 5.5% (95% CI −0.4 to 11.3%). Notably, the number of SSIs was significantly higher in the no-prophylaxis group (5.3% vs. 12.1%; p = 0.010), while no differences were observed in other complications or duration of hospital stay, leading to the conclusion that omitting antibiotic prophylaxis is not recommended [10].

Another study showed no difference in infection rates observed between 24-hour and five-day prophylactic antibiotic regimens for open tibia fractures, suggesting a potentially cost-effective approach with reduced dosage [13]. In particular, in this unblinded randomized controlled trial, patients with Gustilo II open tibia fractures were randomly assigned to receive either 24 hours or five days of prophylactic antibiotics. Results showed that the infection rates were 23% (nine out of 40 patients) in the 24-hour group and 19% (seven out of 37 patients) in the five-day group, with no statistically significant difference between the two groups (p = 0.699), suggesting that a shorter duration of antibiotic prophylaxis may be sufficient for preventing infections in such cases, potentially reducing the risk of antibiotic-related complications and healthcare costs [13].

Overall, while prophylactic antibiotics play a crucial role in preventing infections in emergency surgery, their use should be tailored to the specific clinical scenario, weighing the benefits against potential risks and considering factors such as procedure type, patient population, and optimal duration of administration. Further research, including larger, well-controlled studies, is needed to refine guidelines and optimize antibiotic prophylaxis practices in emergency surgical settings.

Prophylactic Antibiotics in Elective Surgery

A PubMed search conducted within the last 20 years (2004-2024), using the keywords "prophylactic antibiotics," "surgical site infections," and "elective surgery," yielded 215 results. Among these, 25 articles were selected as they met the criteria of being clinical trials or randomized controlled trials, while the remaining were excluded as they were mostly meta-analyses, systematic reviews, or non-relevant prospective studies [11,20,23-25].

Studies on the use of prophylactic antibiotics during elective surgeries to prevent SSIs have revealed conflicting results. In particular, while some studies report significant reductions in SSIs, others find no significant difference between the antibiotic and non-antibiotic groups. The use of prophylactic antibiotics has been shown to significantly reduce infection rates in certain surgical procedures, including cesarean sections, colorectal surgery, breast cancer, and neurosurgical cranial and spinal procedures [11,24-37].

For instance, in a single-blind randomized controlled trial focusing on colorectal surgery, the incidence of SSIs was significantly lower in patients receiving prophylactic antibiotics compared to the control group, with an incidence of 13 out of 157 patients (8.28%) in the experimental group, contrasting with 27 out of 152 patients (17.76%) in the control group [24].

Additionally, in a phase IV randomized controlled trial focusing on breast cancer surgery, prophylactic ampicillin-sulbactam significantly reduced the SSI rate in the prophylaxis group compared to the control group [31]. In this study, a total of 369 patients were included in the final analysis, with 187 allocated to the prophylaxis group and 182 randomly assigned to the control group. Prophylaxis demonstrated a significant reduction in the SSI rate (4.8%) compared to the control group (13.7%), with an RR of 0.35 (95% CI: 0.17-0.73). Moreover, the mean SSI-related cost was substantially higher in the control group ($20.26 USD) compared to the prophylaxis group ($8.48 USD) [31].

In another study examining the dosing regimen of prophylactic antibiotics, a single-dose regimen was associated with a higher incidence of incisional SSIs (27/190 or 14.2%) compared to a three-dose regimen (8/187 or 4.3%). These findings emphasize the effectiveness of prophylactic antibiotics in reducing postoperative infections across various surgical procedures, with three-dose cefmetazole administration being significantly more effective for the prevention of incisional SSI than single-dose antibiotic administration [36].

Similarly, in the context of neurosurgical cranial and spinal procedures, while no statistically significant difference in postoperative infections was observed between groups receiving different prophylactic antibiotics (cefuroxime vs. cefotaxime), notable findings highlighted the significance of administering parenteral antibiotics before surgery [26]. This practice was shown to reduce the incidence of postoperative infections, particularly in cases with increased risk factors (SSI). Notably, patients with a higher ACA score (>2/3), longer surgical intervention durations (>4 hours), contaminated wounds, and comorbidities demonstrated a greater benefit from preoperative antibiotic administration in mitigating the risk of postoperative infections [26].

In elective gynecologic abdominal surgery, the use of prophylactic antibiotics did not show a significant reduction in postoperative infection rates [37]. In this study, 258 women underwent elective gynecologic surgery, and the prophylactic antibiotics used were cefazolin (41.9%), cefoxitin (36.4%), and augmentin (9.7%). However, while the rate of single-dose cefazolin usage did not differ significantly between control and study groups (10.1% vs. 12.4%), postoperative oral antibiotic usage notably decreased (31.8% vs. 14.7%). Despite similar infection rates, administering a single dose of cefazolin to all patients would have significantly reduced antibiotic costs by 91.8%, suggesting that implementing a single dose of a prophylactic antibiotic could substantially reduce antibiotic costs [37].

However, the effectiveness of prophylactic antibiotics varies depending on the type of surgery. Various studies provide evidence that prophylactic antibiotics may not be necessary in certain surgical procedures or patient populations and may not lead to improved outcomes in terms of reducing SSIs [25,27,29,30,33,34,38-40].

For instance, in a study comparing elective laparoscopic cholecystectomy, which was performed on 529 patients, no significant differences in clinical characteristics were found between the antibiotic group (n = 249, cefotetan 1 g, 1 dose/prophylactic) and the non-antibiotic group (n = 260) [25]. Similarly, in another study involving 570 patients who underwent laparoscopic cholecystectomy, there was no statistically significant difference between the groups analyzed with respect to any of the demographic and clinical features analyzed [27]. In this particular study, patients were separated into groups 1 (n = 193), which received physiologic saline as a placebo; group 2 (n = 191), which received a first-generation cephalosporin (cefazolin; 1 g); and group 3 (n = 186), which received a second-generation cephalosporin (cefuroxime axetil, 750 mg). Results have shown that the SSI rate was 1.2% in total, and there was no statistical difference regarding SSI among the three different groups [27]. Furthermore, in another study examining prophylactic antibiotic use in laparoscopic cholecystectomy, low-risk patients were randomly assigned to two groups: 68 patients (group 1) received cefazolin 1 g intravenously after induction of anesthesia, and 76 patients (group 2) were not given prophylactic antibiotics. In group 1, there were three (4.41%) cases of wound infection, three (4.41%) cases of pulmonary infections, and one (1.47%) case of urinary tract infection. In group 2, there were two (2.63%) cases of wound infection, two (2.63%) cases of pulmonary infections, and three (3.95%) cases of urinary tract infection. Again, no significant difference existed in the complication rates, suggesting that the use of prophylactic antibiotics did not decrease the rate of postoperative infection complications and SSIs and was not deemed necessary in low-risk patients undergoing laparoscopic cholecystectomy [34]. Similarly, another study revealed that prophylactic antibiotics in elective laparoscopic cholecystectomy are not recommended as they do not decrease the already low rate of postoperative infectious complications [38]. In particular, at the time of induction of anesthesia, group A patients (n = 141) received 1 g of cefazolin, and group B patients (control; n = 136) received 10 mL of isotonic sodium chloride solution. The overall rate of infection was 1.1% for a total of 277 patients (0.7% for group A patients and 1.5% for group B patients). There were no risk factors contributing to infection complications that could be identified [38].

In another study focusing on laparoscopy for uncomplicated gynecologic conditions, a total of 218 patients were recruited and were divided into two groups. Patients in group A (n = 115) received oral azithromycin 1 g daily for three days (i.e., the day before, the day of, and the day after the procedure), while patients in group B (n = 103) received placebo therapy [30]. Follow-up analysis revealed that antibiotic prophylaxis was not able to achieve a statistically significant reduction in postoperative febrile or infective morbidity [30].

Moreover, in a prospective randomized control study evaluating wound infection rates in 395 patients with various types of hernia undergoing elective mesh repair, 237 patients (60.0%) received prophylactic cefazolin (study group), while the remaining 158 patients (40.0%) did not receive any prophylactic antibiotics (control group) [29]. Patients were monitored for infections at multiple intervals postoperatively, including at 10 days, 30 days, 12 months, and annually for at least two years. The results revealed that eight patients (2.03%) experienced infection at the surgical site, with two (1.27%) occurring in the control group and six (2.53%) in the study group, suggesting that preoperative administration of single-dose cefazolin for prosthetic hernia repairs did not markedly decrease the risk of wound infection [29]. Similarly, in a prospective, randomized, double-blind trial involving 450 patients undergoing primary inguinal hernia repair electively using polypropylene mesh, analysis of 334 out of the 450 patients who completed a one-month follow-up period revealed that the overall infection rate was 8.7% (29 out of 334), with a wound infection incidence of 7% in the antibiotic group and 10.5% in the control group. However, while antibiotic prophylaxis was associated with a decreased incidence of wound infection compared to the control group, this difference was not found to be statistically significant [33].

The effectiveness of prophylactic antibiotics in elective surgeries varies across different surgical procedures and patient populations. While prophylaxis has been shown to reduce infection rates, improve patient outcomes, and potentially lower healthcare resource utilization and costs in some cases, its impact may not be significant in others. Therefore, individualized decision-making based on the specific surgical context and patient characteristics is crucial to optimizing the use of prophylactic antibiotics in emergency surgeries.

Alternative Prophylactic Measures in Emergency and Elective Surgeries

From the studies identified through the PubMed search, only two studies have compared the use of prophylactic antibiotics between emergency and elective surgeries in the past 20 years, one of which is ongoing [11,23]. These two studies offer valuable insights into the efficacy of prophylactic interventions in mitigating postoperative complications across different surgical contexts. The first study, a multi-center randomized controlled trial conducted in five hospitals in Denmark, focused on prophylactic incisional negative pressure wound therapy (NPWT) in obese women undergoing elective or emergency cesarean sections [11]. In this study, 432 women were assigned to receive NPWT, while 444 women had a standard dressing. The results showed that SSIs occurred in 4.6% of women treated with incisional NPWT compared to 9.2% of women treated with a standard dressing, indicating a significant reduction in infection risk with NPWT (RR 0.50, 95% CI 0.30-0.84; number needed to treat 22; p = 0.007). Incisional NPWT also significantly reduced wound exudate [11]. The second study investigates the effectiveness of a vaginal antiseptic wash prior to a cesarean section in reducing postoperative infections [23]. This ongoing study aims to assign women undergoing elective or emergency cesarean sections to either the intervention group (1% povidone-iodine or chlorhexidine) or the control group (no-irrigation) and assess the occurrence of post-cesarean section infection. The results of this study may provide important data to define the future uniform use of vaginal antiseptic wash immediately prior to cesarean section and to determine the best antiseptic wash details for reducing postoperative infections or complications [23]. Moreover, this research will enhance our understanding of the optimal approach to reducing postoperative infections in both elective and emergency surgeries.

Discussion

This comparative analysis underscores the importance of assessing prophylactic antibiotics to effectively mitigate the risk of SSIs in both emergency and elective surgeries, highlighting several key findings and considerations.

In emergency surgery, the administration of prophylactic antibiotics plays a crucial role in mitigating the risk of SSIs amidst the urgency of the procedure. Indeed, it has been shown that timely initiation of antibiotics, tailored antibiotic selection, and rapid intervention in high-risk scenarios contribute to improved patient outcomes. Moreover, studies have demonstrated the effectiveness of prophylactic antibiotics in reducing SSIs in certain emergency procedures, such as cesarean sections and acute calculous cholecystitis [9-11,14,16]. Nevertheless, the necessity of prophylactic antibiotics in all emergency surgeries remains a debated subject, as evidenced by studies showing no significant impact on infection rates in procedures like laparoscopic cholecystectomy for mild to moderate acute cholecystitis [8].

On the other hand, in elective surgery, the benefits of prophylactic antibiotics are more apparent due to meticulous preoperative planning and a controlled environment for administration. In this case, optimal timing, tailored antibiotic regimens, and reduced urgency allow for more effective infection prevention strategies. Studies have reported significant reductions in SSIs in various elective procedures such as colorectal surgery, breast cancer surgery, and neurosurgical cranial and spinal procedures [11,24,26,28,31,36,37]. However, the effectiveness of prophylactic antibiotics may vary depending on the type of surgery, with some studies suggesting that antibiotic prophylaxis may not be necessary in certain low-risk elective procedures like laparoscopy for uncomplicated gynecologic conditions, laparoscopic cholecystectomy, or hernia repair surgeries, similar to findings in emergency surgery cases [25,27,29,30,33,34,38-40].

Overall, comparing the use of prophylactic antibiotics between emergency and elective surgeries reveals nuanced differences in their efficacy and impact on patient outcomes. While prophylactic antibiotics are essential in both contexts, their optimal use requires careful consideration of factors such as procedure type, patient population, and risk factors for SSIs. Importantly, the use of prophylactic antibiotics needs to be tailored to specific surgical procedures and patient populations. The rightful use of these antibiotics can potentially reduce the incidence of SSIs, leading to significant savings in healthcare resource utilization as well as a reduction in hospital stays. Moreover, it has been shown that unnecessary antibiotics could lead to an increased risk of antibiotic resistance and unnecessary costs, which can be avoided [11,24-26,35]. Also, reducing the dosage of antibiotics has shown no discernible difference in terms of SSIs, potentially offering opportunities for cost reduction [13,32].

Limitations of the analysis include the inherent variability in study designs, patient populations, and surgical practices across different settings, which may affect the generalizability of findings. Additionally, the analysis relied on the available literature at the time of review and may not capture the most recent developments in the field.

Overall, this methodology aimed to provide a systematic and evidence-based comparison of the benefits of prophylactic antibiotics in emergency and elective surgeries to inform clinical practice and healthcare policy decisions.

## Conclusions

Prophylactic antibiotics play a crucial role in preventing SSIs in both emergency and elective surgeries. While the benefits are evident in both scenarios, the optimal approach may differ. Further research is needed to refine guidelines and optimize antibiotic prophylaxis practices in both emergency and elective surgical settings. In particular, studies comparing different antibiotic regimens, dosages, and administration protocols can provide valuable insights into the most effective strategies for infection prevention across diverse surgical contexts. Additionally, ongoing research investigating novel interventions such as vaginal antiseptic washes may offer promising approaches to further enhance surgical outcomes and reduce the incidence of SSIs in both emergency and elective surgeries.
