# Correction: A Comparative Analysis of Prophylactic Antibiotic Administration in Emergency Surgery Versus Elective Surgery: A Comprehensive Review

**DOI:** 10.7759/cureus.c359

**Published:** 2025-10-13

**Authors:** Christopher R Meretsky, Brandon Krumbach, Anthony T Schiuma

**Affiliations:** 1 Surgery, St. George's University School of Medicine, Great River, USA; 2 Surgery, St. George’s University School of Medicine, Great River, USA; 3 Orthopedic Surgery, Holy Cross Hospital, Fort Lauderdale, USA

This article has been corrected to fix the following typos in the PRISMA diagram and article text:

- Record totals from both searches are now included in the PRISMA diagram
- In the first paragraph following the '*Prophylactic Antibiotics in Elective Surgery' *header, '45' has been corrected to '25'

